# Four Decades of Forest Persistence, Clearance and Logging on Borneo

**DOI:** 10.1371/journal.pone.0101654

**Published:** 2014-07-16

**Authors:** David L. A. Gaveau, Sean Sloan, Elis Molidena, Husna Yaen, Doug Sheil, Nicola K. Abram, Marc Ancrenaz, Robert Nasi, Marcela Quinones, Niels Wielaard, Erik Meijaard

**Affiliations:** 1 Center for International Forestry Research, Bogor, Indonesia; 2 Centre for Tropical Environmental and Sustainability Science, School of Marine and Tropical Biology, James Cook University, Cairns, QLD, Australia; 3 Department of Ecology and Natural Resource Management (INA), Norwegian University of Life Science (NMBU), Ås, Norway; 4 Durrell Institute of Conservation and Ecology, University of Kent, Canterbury, Kent, United Kingdom; 5 Borneo Futures project, People and Nature Consulting International, Ciputat, Jakarta, Indonesia; 6 Sabah Wildlife Department, Kota Kinabalu, Sabah, Malaysia; 7 HUTAN, Kinabatangan Orang-utan Conservation Programme, Kota Kinabalu, Sabah, Malaysia; 8 North England Zoological Society, Chester Zoo, Chester, United Kingdom; 9 SarVision, Wageningen, The Netherlands; 10 School of Biological Sciences, University of Queensland, Brisbane, QLD, Australia; University of Massachusetts, United States of America

## Abstract

The native forests of Borneo have been impacted by selective logging, fire, and conversion to plantations at unprecedented scales since industrial-scale extractive industries began in the early 1970s. There is no island-wide documentation of forest clearance or logging since the 1970s. This creates an information gap for conservation planning, especially with regard to selectively logged forests that maintain high conservation potential. Analysing LANDSAT images, we estimate that 75.7% (558,060 km^2^) of Borneo's area (737,188 km^2^) was forested around 1973. Based upon a forest cover map for 2010 derived using ALOS-PALSAR and visually reviewing LANDSAT images, we estimate that the 1973 forest area had declined by 168,493 km^2^ (30.2%) in 2010. The highest losses were recorded in Sabah and Kalimantan with 39.5% and 30.7% of their total forest area in 1973 becoming non-forest in 2010, and the lowest in Brunei and Sarawak (8.4%, and 23.1%). We estimate that the combined area planted in industrial oil palm and timber plantations in 2010 was 75,480 km^2^, representing 10% of Borneo. We mapped 271,819 km of primary logging roads that were created between 1973 and 2010. The greatest density of logging roads was found in Sarawak, at 0.89 km km^−2^, and the lowest density in Brunei, at 0.18 km km^−2^. Analyzing MODIS-based tree cover maps, we estimate that logging operated within 700 m of primary logging roads. Using this distance, we estimate that 266,257 km^2^ of 1973 forest cover has been logged. With 389,566 km^2^ (52.8%) of the island remaining forested, of which 209,649 km^2^ remains intact. There is still hope for biodiversity conservation in Borneo. Protecting logged forests from fire and conversion to plantations is an urgent priority for reducing rates of deforestation in Borneo.

## Introduction

Conservationists have historically prioritized the protection of ‘pristine’, ‘old-growth’ tropical forests over human-modified ones [1]. Pristine tropical forests are becoming increasingly rare, however, particularly in the lowlands of South-East Asia [2] due to widespread timber extraction (“logging”), conversion to other land uses and increased vulnerability to fire. The conservation value of selectively-logged forests has been increasingly highlighted as requiring recognition [3]–[5]. Selective timber extraction prevails in tropical forests meaning that only a few stems (typically 4–10) are removed from each hectare leaving a diverse forest still standing [5]–[10]. These modified habitats retain appreciable biodiversity [3], [5], [9] and serve as effective buffers and corridors for wildlife moving between intact forest fragments [11].

Borneo's forests include old-growth lowland, hill and montane dipterocarp forests, freshwater and peat swamp forests, heath forests (*kerangas*), and mangrove forests (including areas dominated by the palm *Nypa fruticans Wurmb* locally termed *nipah*) [12]. These forests possess some of the richest biological communities on the planet and should therefore be preserved [12]. But, much has already been logged [13] − between 1980 and 2000 more round wood was harvested from Borneo than from Africa and the Amazon combined [14] − or destroyed by fire [15], [16], or converted to plantations. Forest conversion encompasses clearing forest to establish industrial oil palm (*Elaeis guineensis*) [17], and to a lesser extent acacia (*Acacia* spp) and rubber tree (*Hevea brasiliensis*) plantations [17], [18]. There is no island-wide satellite-based documentation of forest clearance, conversion or logging and the island remains under studied in this respect compared to other major tropical regions, although a recent study filled this gap for the northern part of Borneo [19]. Confusion reigns over the actual extent of deforestation, remaining intact and logged-over forests hampering proper conservation planning. For example, Indonesia's pledge to maintain at least 45% of forest in the Indonesian part of Borneo (Kalimantan) was criticized by environmental groups reporting that Kalimantan retains only 30% forest cover [20], appreciably less than the 55% reported by Indonesia's Ministry of Forestry [21].

Building on a detailed, LANDSAT-based, spatial inventory of forest cover, clearance and logging over the 1973–2010 period, the era of industrial-scale forest exploitation on the island [13], we address the following questions: (i) what was the extent of forest cover in the early 1970s; (ii) how much has been selectively logged or cleared since; and (iii) how are remaining intact and logged forests distributed across zones designated for protection, timber production, or conversion to plantations?

## Methods

### Overall approach

We mapped forest extent, and deforestation for the period 1973–2010 as well as total logged area since 1973 over the whole island of Borneo (737,188 km^2^) at medium spatial resolution (pixel size  = 60 m×60 m). To assess deforestation (forest clearance) we developed a baseline forest cover map for the year 1973 using LANDSAT MSS imagery (pixel size  = 60 m×60 m). Combining our 1973 baseline map with a 2010 forest cover map (pixel size  = 50 m×50 m) developed using ALOS PALSAR radar satellite imagery [22] we produced a map of deforestation between 1973 and 2010 (pixel size  = 60 m×60 m). To estimate the area of forest converted to plantations since 1973 and to correct for the tendency of the ALOS PALSAR classification to confound mature plantations for forest [22], we mapped all industrial oil-palm and timber plantations as of 2010 using LANDSAT imagery. Finally, using LANDSAT imagery and a MODIS-based percent tree cover map, we mapped the total logged-over forest area from 1973 to 2010 using logging roads as a proxy. The following sections elaborate the datasets (Table 1) and associated methods.

**Table 1:** Summary of Spatial Data.

Description	Source Data	Spatial resolution	Classification Method	Years of observation
1973 Forest cover	LANDSAT MSS	60 m×60 m	Supervised classification followed by visual interpretation	1973
2010 Forest cover	ALOS-PALSAR [22]	50 m×50 m	Unsupervised classification	2010
Forest clearance (1973–2010)	LANDSAT MSS and ALOS-PALSAR	60 m×60 m	map comparison and visual interpretation	1973–2010
Total logged area since 1973	LANDSAT MSS, TM, ETM+ imagery; MODIS [35], [36]	Vector data resampled to 60 m×60 m	Visual interpretation and buffering	1973, 1990, 2000, 2000, 2010
Industrial oil-palm (IOPP) and timber plantations (ITP)	LANDSAT MSS, TM, ETM+,	Vector data resampled to 60 m×60 m	Visual interpretation	1973, 1990, 1995, 2000, 2005, 2010

### Mapping of natural forest cover in 1973

**Figure 1:**
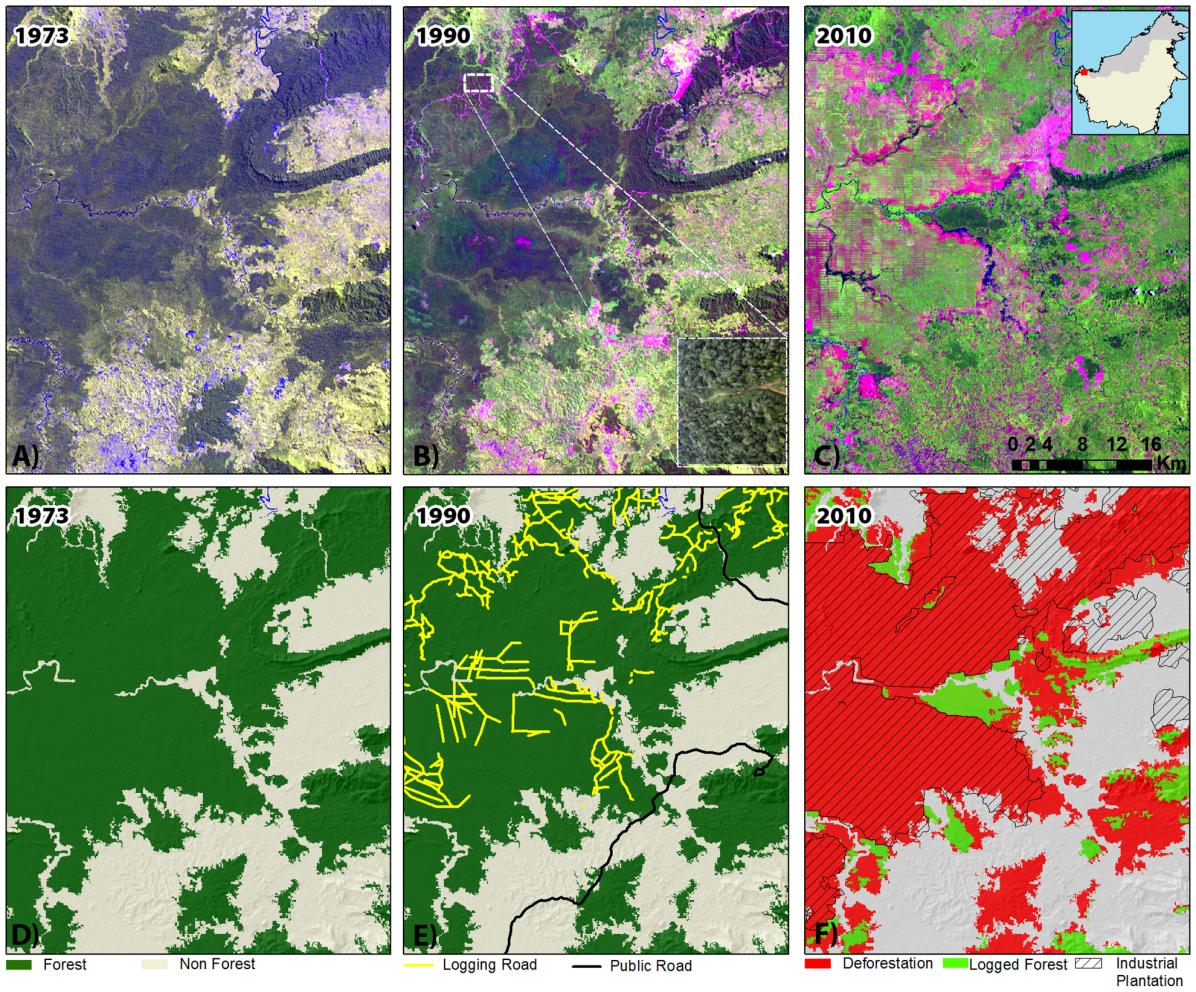
A close-up view of an area in West Kalimantan province, Borneo (see top-right inset for location). In this example, an intact forest in year 1973 was logged in 1990 and became converted to an industrial oil Palm Plantation in 2010. On the 1973 LANDSAT MSS imagery (false color composite: 3-4-2) forest appears dark green (panel A). The resulting forest non-forest supervised classification is shown in panel D. On the 1990 LANDSAT TM imagery (false color composite: 5-4-3) primary logging roads are seen carving through the forest (panel B). The logging roads (yellow lines) and public roads (black lines) are shown in panel E. An aerial view of a primary logging road (Photo by R. Butler [53]) is shown the inset of panel B. On the 2010 LANDSAT TM imagery), the forest has become converted to an industrial oil palm plantation (grid-like patterns). The boundary of the plantations was digitized by visual interpretation (Panel F).

To map 1973 forest cover we submitted 61 LANDSAT MSS images acquired during the 1970s and spanning the whole Borneo to a supervised tree-based classification algorithm [23] that iteratively classified ‘Forest’, ‘Non-forest’, ‘Cloud’, ‘Cloud shadow’, and ‘Water’ classes. All LANDSAT MSS imagery was viewed as a band 4-3-2 (or 3-4-2) false-color composite (Figure 1A).

We obtained 37 of the 61 images from the USGS Global Land Survey (GLS) 1970s collection of ortho-rectified LANDSAT MSS Imagery [24] and completed with 24 additional non-GLS LANDSAT MSS images (obtained from the USGS LANDSAT MSS archive) wherever available to reduce the area contaminated by clouds. The list of LANDSAT MSS images (n = 61) used to map forest cover in 1973 is provided in Table S1 in File S1. Persistently cloudy areas in the 1970s that were observed as ‘Forest’ in 2010 were reclassified as ‘Forest’ in 1973 accordingly. Areas classified as ‘Cloud’ in 1973 and ‘Non-forest’ in 2010 accounted for <5% of Borneo's area. LANDSAT images not sourced from the GLS collection (non-GLS) were geometrically registered to the reference GLS imagery using a second or third order polynomial co-registration technique (RMS<1 pixel), but they were not ortho-rectified because precise data required for ortho-rectification (sensor angle, viewing angle, distance to object) for each individual Landsat image were often not available. Therefore a moderate horizontal displacement of high-elevation features (e.g., mountain peaks) from their actual coordinates might persist. The 61 images were acquired between 1972 and 1980, but because >80% of Borneo's area was imaged between 1972 and 1973, a weighted average by area takes 1973 as the nominal map year (see Figure S1a in File S1).

Measures were taken to ensure high accuracy of the finished 1973 forest cover map, in particular manually editing complex areas such as those obscured by haze or in steep topography, where the supervised classification algorithm often produced classification errors, but where a trained remote sensing analyst could still visually interpret the imagery. Two analysts only were involved as the use of multiple interpreters can compromise the consistency of the results [25].

### Mapping deforestation: 1973–2010

To map deforestation between 1973 and 2010 we compared our baseline map with the 2010 forest cover map developed by SARVISION using ortho-rectified ALOS PALSAR radar imagery [22]. The SARVISION 2010 map distinguishes several forest types by altitude, soil type (peat and mineral), and condition. We collapsed select classes to constitute a general 2010 ‘Forest’ class comparable to our 1973 ‘Forest’ class. Classes within the SARVISION 2010 ‘Forest’ class included tropical lowland forest, tropical mountain forest, freshwater and peat swamp closed and pole forest, riverine forest, mangrove forest and *nipah* mangrove forest. All other classes (e.g., towns, agricultural areas, open-cast mines, water ways) were classified as ‘Non-forest’.

We took all possible measures to check for other discrepancies between the ALOS PALSAR-based 2010 forest classification and our interpretation of what constitutes ‘Forest’ and ‘Non-forest’ on the LANDSAT imagery. In particular, numerous visual checks ensured that small-scale deforestation patches were adequately captured in the final deforestation map by visually reviewing the entire LANDSAT database from 1972–2010 (n = 268). The list of all LANDSAT images (MSS, TM, ETM+) used in this study is provided in Table S1 in File S1. All LANDSAT TM and ETM+ imagery was viewed as a band 4-5-3 (or 5-4-3) false-color composite (Figure 1B&C).

All industrial plantations that existed in year 2010 were digitized via visual interpretation of 268 LANDSAT scenes from the 1970s, 1990, 2000 and 2010 (see Table S1 in File S1). The inspection of multiple images prior to 2010 ensured that those plantations that were mature in a given year and therefore difficult to detect in that year were fully accounted for (cf. [17]). Plantations were identified as large geometrically-shaped areas with distinctive, grid-like or contour-like patterns and homogeneous spectral signatures characteristic of monocultures or recently-cleared lands (Figure 1C&F). In instances where the 2010 ‘Forest’ map from SARVISION overlapped with our LANDSAT-derived 2010 ‘Industrial Plantation’ layer described below, the area of overlap was re-classified as ‘Industrial plantation’ accordingly. Areas that were ‘Forest’ in the 1973 map and ‘Non-forest’ in 2010 were classified as ‘Forest clearance’.

### Mapping logged-over forest: 1973-2010

We estimated the approximate extent of forests impacted by industrial-scale mechanized logging between 1973 and 2010 by first mapping all primary logging roads (large enough to be detected on MSS or TM images), next determining a distance from logging roads beyond which forest extraction typically extends, and then ‘buffering’ roads by this distance. A review of a satellite-based methods of estimating the extent of selectively-logged tropical forests finds that studies which similarly mapped and buffered logging roads [25]–[30] did so effectively, particularly at large spatial scales at which more complex approaches are challenging, inappropriate, or impossible [31].

We digitized the extent of primary logging roads by visually analyzing our 268 LANDSAT images acquired over 1972–2010 (see Table S1 in File S1). Wide logging roads were readily detectable in the LANDSAT imagery (Figure 1B&E). We were capable to detect logging roads under most areas of persistent haze, by zooming in closely and applying a local contrast enhancement, and by digitizing logging roads underneath haze by mouse-click. The expansion of the road network overtime was observed for *c.*1973, 1990, 2000, and 2010. Imagery acquired a year or two before and after these nominal years served to reduce cloud contamination. We also inspected imagery from ca. 1995 and 2005 to better detect disused logging roads less visible due to rapid forest regrowth.

Similarly to our approach for mapping industrial plantations LANDSAT 5&7 (TM and ETM+) images were viewed as band 4-5-3 (or 5-4-3) false color composites enhanced to optimize road detection. Likewise, LANDSAT MSS images were viewed as band 4-3-2 (or 3-4-2) (Figure 1A, B&C). We used ancillary public-road maps from the Indonesian Ministry of Public Works [32] and the Sabah-based NGO HUTAN for Sarawak and Sabah to help distinguish unpaved public roads from logging roads.

We defined our buffer as the maximum distance from primary logging roads at which median measures of percent tree cover measured by the MODIS Vegetation Continuous Field product [33] exhibited depressed values indicative of the effects of logging on canopy cover. Forests within this distance were considered logged. The MODIS dataset defines percent tree cover continuously per pixel across Borneo over twelve consecutive months from March 2000 at 230-metre pixel resolution. To ensure that observations of the relationship between tree cover and distance from roads reflected only the effects of contemporaneous logging, we confined our observations to areas within 2.5 km of logging roads detected in 2000 that were still forested in 2010 and that were at least 5 km from logging roads mapped in 1970, in 1990, or in 2000 where in the latter case they were deforested by 2010. We analyzed an area of 38,940.6 km^2^ surrounding 16,336.4 km of selected logging roads (see Figure S2; Table S2 in File S1) and observed the relationship separately for Kalimantan, Sarawak, and Sabah based on the assumption that logging practices may differ between regions.

Finding the relationship between percent tree cover and distance from roads to be similar between Kalimantan, Sarawak and Sabah, we nominated a single distance threshold and buffered all logging roads by this distance to yield an initial estimate of the total area logged since 1973. This initial estimate was considered conservative, however, given its linear geometry and the fact that imagery detects only relatively marked instances of canopy damage [34]. To address this caveat, we delimited the 2010 forest areas enclosed by the buffered logging roads, and reclassified these ‘enclosed’ forests as logged wherever they met the following criteria: (i) area <100 ha; or ii) felling within the secondary-forest (*hutan sekunder*) class mapped by the Indonesian Ministry of Forestry (MoF) in 2009 and 2000 via visual interpretation of LANDSAT imagery [35], [36]. The MoF secondary-forest class encompasses forests where logging concessions were granted, evidence of logging was observed (e.g. roads), and/or forest perturbation is known to exist, and it is taken generally as the official, broad estimate of logged-over forest. The combined extent of buffered roads and enclosed forests meeting the criteria represents a better approximation of the variable local shape/extent of logged areas. We therefore adopted this combined extent as the ‘total area logged since 1973’. The intersection of this area with 2010 ‘Forest’ in turn defines the extent of ‘Logged forest’ as of year 2010.

### Map validation

**Figure 2:**
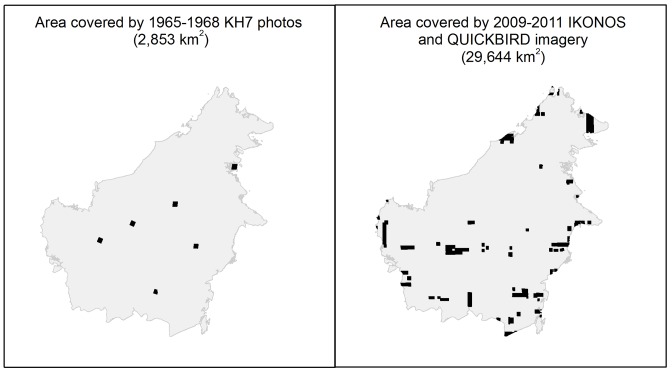
Map showing the areas where validation of the forest and plantation maps was performed. Area covered by 1965–68 KH7 imagery (Black) used to validate the 1973 forest map in Left Panel. Area covered by 2010 IKONOS and QUICKBIRD imagery (Black) used to validate the 2010 forest and plantation maps in Right Panel.

We conducted validations of all our maps to assess accuracy. For the 1973 forest map, we validated the accuracy by comparing it to photos taken from the declassified KH7 satellite-borne camera purchased from [24], having a 1 m^2^ resolution able to discriminate individual tree crowns. The KH7 images were acquired during 1965–1968, i.e., 5–8 years prior to our LANDSAT-MSS 1973 forest cover map. The KH7 images were geometrically registered to our orthographically rectified Global Land Survey (GLS) images using a second or third order polynomial co-registration technique. These photos cover 2,853 km^2^ across six different sites encompassing a variety of topographic conditions (Figure 2). We randomly sampled 322 validation points within this area, with points being at least 1,000 m from each other to minimize spatial autocorrelation [37]. For each point, a 60 m×60 m area was visually interpreted as either ‘Forest’ or ‘Non-forest’ in the KH7 photos at 1∶1,000 scale (see Figure S3 in File S1). A confusion matrix [38], [39] determined the frequency of class agreement between our reference photos and forest map, as determined by overall accuracy and user's and producer's accuracy [40].

We checked the accuracy of the combined 2010 forest and industrial plantation map using Google Earth's collection of high-resolution QUICKBIRD and IKONOS images (0.6–1 m^2^ resolution) accessed using the “Open Layer plug-in” of the open-source software QUANTUM GIS [41]. The total area of these images acquired during 2009–2011 in Borneo spanned 29,644 km^2^ (Figure 2), within which we randomly sampled 1,921 points separated by more than 1000 m. As for the 1973 validation, we visually interpreted each 60 m×60 m area centered on a point to be ‘Forest’, ‘Industrial oil palm plantation (IOPP)’, Industrial timber plantation (ITP)’ or ‘Non-forest’ in the high-resolution imagery. Several QUICKBIRD validation points are shown in Figure S4 (in File S1). We determined the frequency of class agreement between these reference points and our 2010 combined forest and plantation map using a confusion matrix.

## Results

### Validation of Maps

The overall accuracy for the 1973 ‘Forest’ and ‘Non-forest’ classification is 89%. The per-class accuracies of ‘Forest’ and ‘Non-forest’ were similarly high, with user's accuracy ranging from 82–92%, and producer's accuracy ranging from 85–91% (Table 2; Table S3 in File S1). The overall accuracy for the 2010 map of ‘Forest’, ‘Non-forest’, ‘Industrial oil palm plantation (IOPP)’, and ‘Industrial timber plantation (ITP)’ is 93% (Table 3, Table S4 in File S1). The per-class accuracies of those four land cover classes were similarly high, with user's accuracies ranging from 83–97% and producer's accuracies from 75–94%. The lowest accuracy (75%) is for the ITP class, as there were few validation points for this class (n = 37).

**Table 2:** Accuracy results of 1973 ‘Forest’, and ‘Non-forest’ classification.

Class	User Accuracy	Producer Accuracy
Forest (n = 210)	92.3%	90.6%
Non-forest (n = 113)	82.3%	85.3%

Overall accuracy = 89.0%.

**Table 3:** Accuracy results of 2010 ‘Forest’, ‘Non-forest’, ‘Industrial oil palm plantation (IOPP)’, and ‘Industrial timber plantation (ITP)’ classification.

Class	User Accuracy	Producer Accuracy
Forest(n = 458)	91.4%	90.3%
Non-forest (n = 1208)	95.4%	94.6%
IOPP (n = 218)	83.0%	94.7%
ITP (n = 37)	97.2%	75%

Overall accuracy = 93.1%.

### Forest Clearance

**Figure 3:**
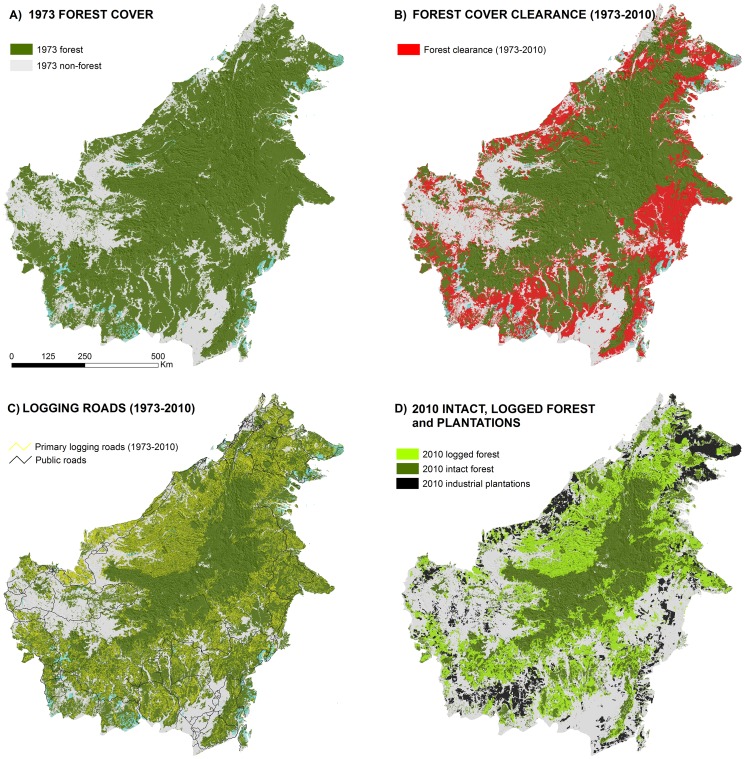
Four decades of forest persistence, clearance and logging on Borneo. Forest (dark green) and non-forest (white) in year 1973, and residual clouds (cyan) in Panel A. Areas of forest loss during 1973–2010 (red) in Panel B. Primary logging roads from 1973–2010 (yellow lines) in Panel C. Remaining intact forest (dark green), remaining logged forest (light green), and industrial oil palm and timber plantations (Black) in year 2010 in Panel D.

We estimate that in 1973, 75.7% of Borneo remained under natural forest. That is 558,060 km^2^ of mainly intact (i.e., unlogged) old-growth forest (Figure 3A; Table 4). By 2010, this forest area had been reduced by 168,493 km^2^, representing a 30.2% forest loss over the previous four decades (Figure 3B). More than 97% (164,644 km^2^) of this deforestation occurred in Borneo's coastal lowlands (<500 m asl). The Sultanate of Brunei and Sarawak have the lowest rates of deforestation with 8.4% and 23.1%, respectively. Sabah and Kalimantan have the highest rates (39.5% and 30.7%) (Table 4). Of the 168,493 km^2^ total forested area lost since 1973, 51% (86,339 km^2^) had been logged between 1973 and 2005, and 33% (56,080 km^2^) had been converted to industrial plantations (IOPP and ITP). In 2010, the area planted in IOPPs and ITPs was 64,943 km^2^ and 10,537 km^2^, respectively, representing 10% of Borneo (Figure 3D, Table S5 in File S1).

**Table 4:** Intact, logged and cleared forest area by country and elevation, 1973 and 2010.

	Borneo	Brunei	Kalimantan	Sabah	Sarawak
Total land area (km^2^)					
0–500 m	601,276	5,584	446,995	54,371	94,326
501–1000 m	98,404	185	62,705	14,379	21,135
1001–1500 m	32,836	26	21,154	4,217	7,439
1501–2000 m	4,504	1	2,549	865	1,089
>2000 m	168	0	19	133	16
*All elevations*	*737,188*	*5,796*	*533,422*	*73965*	*124,005*
Forest area in 1973 (km^2^)					
0–500 m	424,753	4,286	318,050	39,721	62,697
501–1000 m	96,159	184	61,811	13,220	20,944
1001–1500 m	32,504	26	21,118	3,950	7,411
1501–2000 m	4,481	0	2,544	852	1,085
>2000 m	163	0	19	129	16
*All elevations*	*558,060*	*4,496*	*403,541*	*57,871*	*92,152*
Intact Forest area in 2010 (km^2^)					
0–500 m	114,017	3,100	95,123	7,344	8,450
501–1000 m	65,036	178	55,560	4,279	5,019
1001–1500 m	26,552	26	20,844	1,828	3,855
1501–2000 m	3,894	0	2,504	568	821
>2000 m	151	0	17	119	16
*All elevations*	*209,649*	*3,303*	*174,048*	*14,138*	*18,161*
Logged Forest area in 2010 (km^2^)					
0–500 m	146,092	808	99,609	11,634	34,042
501–1000 m	27,531	7	5,633	7,015	14,876
1001–1500 m	5,710	0	269	1,941	3,500
1501–2000 m	573	0	40	270	263
>2000 m	11	0	2	9	0
*All elevations*	*179,917*	*815*	*105,553*	*20,868*	*52,681*
Forest area loss 1973–2010 (km^2^)					
0–500 m	164,644	378	123,318	20,743	20,205
501–1000 m	3,593	0	618	1,926	1,048
1001–1500 m	241	0	5	181	56
1501–2000 m	14	0	0	14	0
>2000 m	1	0	0	1	0
*All elevations*	*168,493 (30.2%)*	*378 (8.4%)*	*123,941 (30.7%)*	*22,865 (39.5%)*	*21,309 (23.1%)*

### Logging roads and logged forests

We calculated that 271,819 km of primary logging roads were opened between 1973 and 2010 (Figure 3C), equating to an overall density of 0.48 km road per km^2^ of forest in 1973 (Table 5). The highest density is found in Sarawak (0.89 km km^−2^) and the lowest density in Brunei (0.18 km km^−2^).

**Table 5:** Length and density of primary logging roads by country.

	1973 forest cover (km^2^)	Logging road length (km)	Logging road density (km/km^2^)
Brunei	4,496	818	0.18
Kalimantan	403,541	151,101	0.37
Sabah	57,871	37,660	0.65
Sarawak	92,152	82,239	0.89
**Borneo**	**558,060**	**271,819**	**0.48**

**Figure 4:**
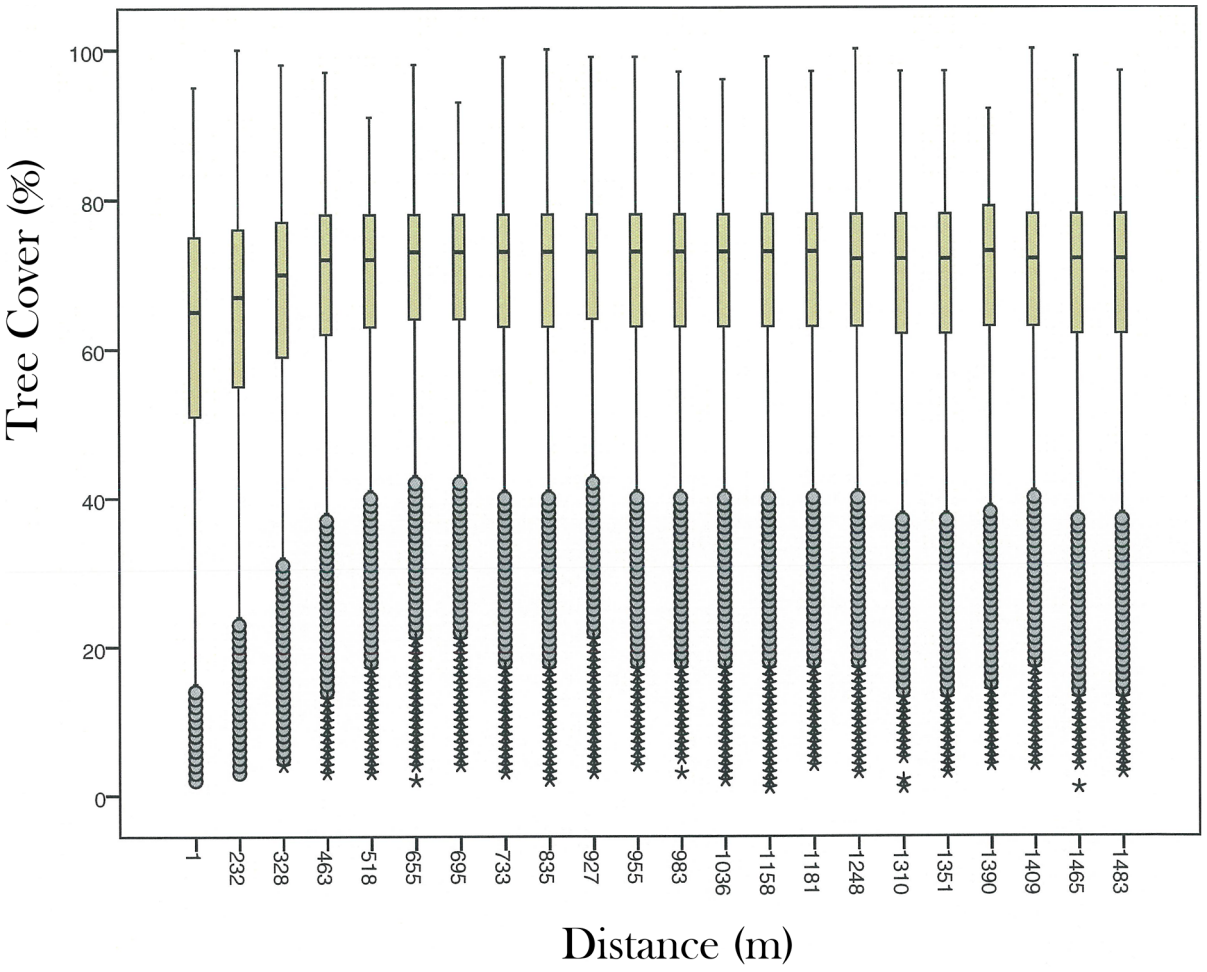
Relationship between distance to selected logging roads established in year 2000 and percent tree cover (2000/2001). The black points at approximately 70% on the y-axis are median values for each distance class. The boxes centered on each median are bounded by the 75^th^ and 25^th^ percentile values of each distance class. Grey circles flag ‘outliers’ having values lower/higher than the 25^th^/75^th^ percentile by 1.5–3 times the inter-quartile range. Asterisks flag extreme ‘outliers’ having values >3 times lower/higher than the 25^th^/75^th^ percentile value.

Percent tree cover exhibited depressed values indicative of the effects of logging on canopy cover at least 460 m nominally from primary logging roads in Borneo (Figure 4). This nominal distance is measured from pixel center to pixel center and may be extended to 700 m practically upon accounting for the breadth of pixels and variation in where a logging road may fall therein. Taking 700 m as the upper distance thresholds and then applying our empirical 700 m buffer distance to all logging roads (Figure 3C) and reclassifying any enclosed forests as ‘Logged forest’, we estimate the total forest area logged since 1973 at 266,257 km^2^. Of these 266,257 km^2^ some 67.5% (179,917 km^2^) remain standing as forest in 2010, 32.5% (86,340 km^2^) were cleared.

### Remaining intact forest in 2010

An estimated 389,566 km^2^ (52.8%) of the island remained forested (either intact or logged) in 2010. Intact forests represent 53.8% (209,649 km^2^) of the total remaining forest area or 28.4% of the whole Borneo (Table 4). Brunei has the highest proportion of intact forest area at 56.9%, compared to 32.6% in Kalimantan, 19.1% in Sabah and 14.6% in Sarawak.

Much of the remaining intact forests will be logged and converted under the current forest-use designations. Some 42% (88,150 km^2^) of intact forests fall within the ‘production forest’ land-use class and will be logged (Table S5 in File S1). The actual area of intact forest in the land use designated for production is greater than the area of intact forests in protected areas (Table S5 in File S1). A further 16% (33,548 km^2^) of these intact forests will be converted based on their intended land-use. Given the present distribution of intact forest across land use designations, the future extent of intact forest for all Borneo may decline to only 87,953 km^2^, or 11.9% of Borneo, assuming that all production forests are ultimately logged, area designated for conversion is ultimately converted or degraded, assuming protected intact forests remain unperturbed and that the extents of these land-use decisions remain unchanged. For the lowland forests (<500 m asl), the most threatened, the corresponding figures are 33,773 km^2^ and 5.6% of Borneo's lowlands.

We observed an appreciable area of logged forest within the protection designation, which prohibits logging. Of the 179,917 km^2^ of current logged forest in Borneo (Table 4), 10.3% falls within the protected areas (Table S5 in File S1) wherein it constitutes 17% of all forest cover. Some, but not all, of this logged forest is evidence of illegal logging. In other cases, it evidences legally logged forests that were later re-designated for protection, e.g., the Sebangau National Park, declared in 2004 [42]. While our data do not allow distinguishing illegal from legal logging *per se*, it is reasonable to assume that some logged forests within the protection designation were exploited illegally.

### Spatial and Temporal Trends in Logging, 1973–2010

**Figure 5:**
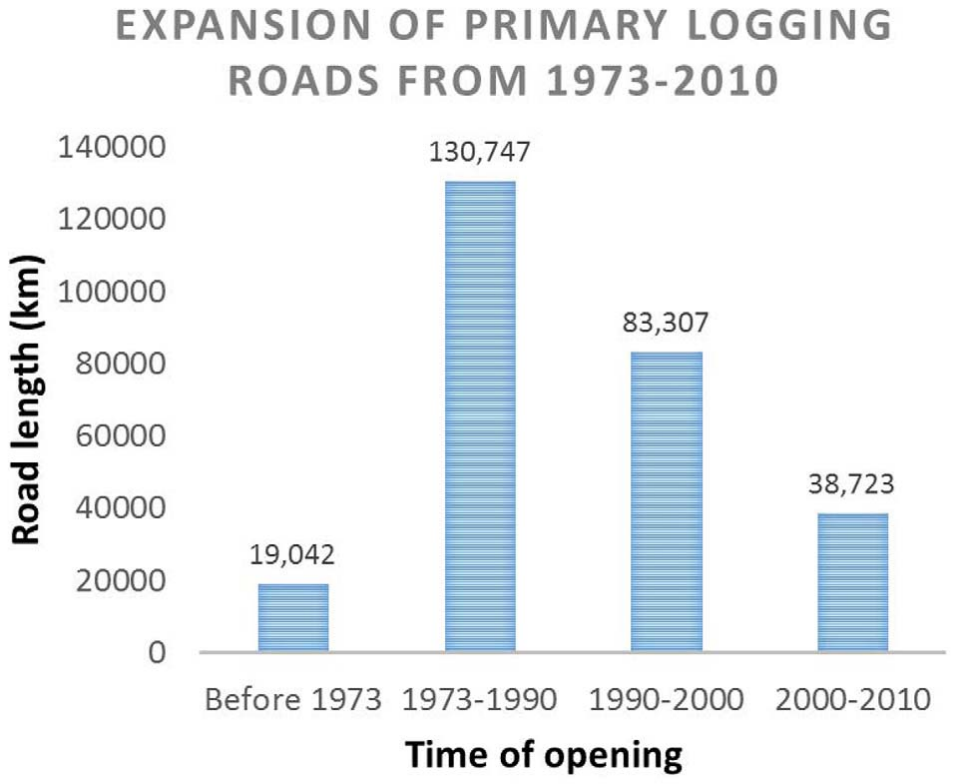
Expansion of the primary logging road network in Borneo from 1973 until 2010.

Temporal and spatial trends in logging describe an initial ‘boom’ in the exploitation of easily accessible lowland forests, followed by a slower expansion into remaining, more marginal forests abutting highland protected forests. Over half of all logging roads by length were established prior to 1990 (Figure 5), when the logging industry was relatively young and intact forests were easily accessible.

**Figure 6:**
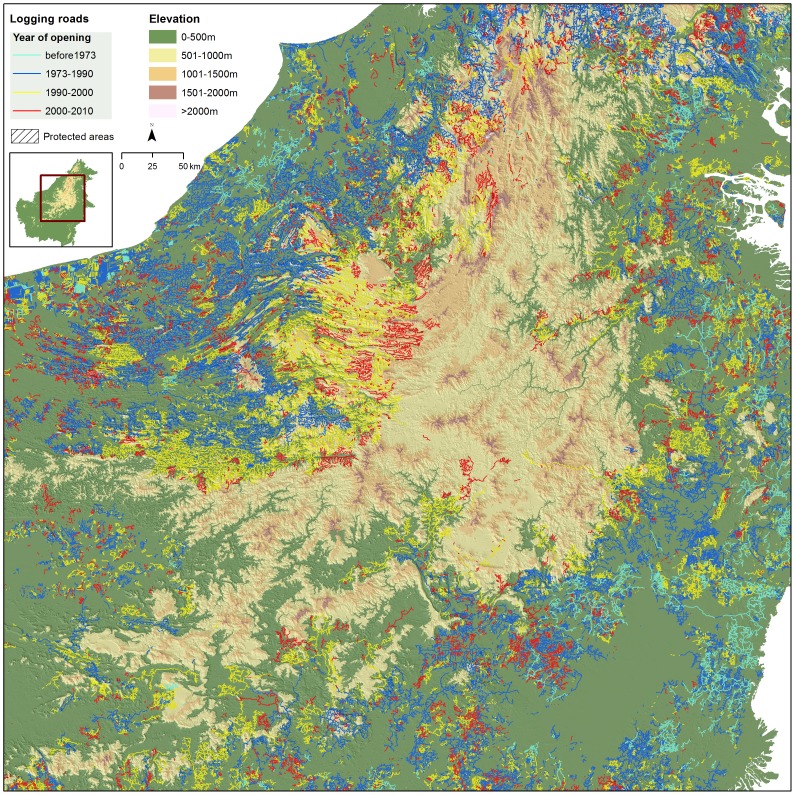
The heart of Borneo and the spatial progression of logging since 1973 depicting a logging ‘frontier’ moving steadily upward and inland, from the lowland coasts to the highlands. In many areas, logging roads are surrounding and abutting against the edges of highland protected forests – the last contiguous bastions of intact forest.

Since 2000 the rate of logging roads expansion has approximately halved (Figure 5), reflecting a growing scarcity of unlogged, accessible forests. The spatial progression of logging since 1973 therefore depicts a logging ‘frontier’ moving steadily upward and inland, from the lowland coasts to the highlands, in search of remaining unlogged forests (Figure 6). In many areas, logged forests have expanded to surround and abut against the edges of highland protected forests – the last contiguous bastions of intact forest.

## Discussion

### Methodological considerations

Our study has generated the first full island-wide maps of forest cover and forest cover change in Borneo since industrial logging began in early 1970s, with high classification accuracies, appropriate for analyzing fine scale changes in forest cover [43]. We note that >90% of the validation area (the black areas in Figure 2) is distributed in the lowlands (between 0–500 m asl), but the lack of validation area in mountains of the island did not overestimate our validation because forest cover loss occurr**e**d in the lowlands (0–500 m). Nevertheless, our accuracy assessments were conducted on just a 0.4–4% spatial subset, and therefore may vary across the study area.

We caution that our estimates of remaining logged forest area are likely an under-estimation because they did not capture most narrow, ephemeral, sub-canopy logging trails branching off the main logging roads or smaller-scale community-level logging activities. For example, the small trails created by illegal logging such as those found in Gunung Palung national park in 1999 [14] remained undetected by our method. In turn, this means that our remaining intact forest area may be over-estimated. A direct verification of this over-estimation is not possible because of a lack of contemporary, fine-scale annual satellite imagery in which to observe logging trails. We observe a general agreement between our empirically-estimated buffer distance of 700 m − the distance from logging roads within which timber extraction extends − and similar observations in the literature. It compares favorably with estimates of 350–1000 m in Sabah and Sarawak [19], <500 m in Papua New Guinea [44], and 1 km in Central Africa [27] and Sumatra [25]. Finally, we acknowledge the limitations of buffering primary logging roads with a fixed distance to estimate the extent of logged forests because this buffer distance varies with accessibility such as topography and soil type. Modeling the buffer distance across landscapes using spatial attributes is an area of research where we would hope to make further refinements.

Our results merit comparison with those from an earlier study of forest degradation in Malaysian Borneo. Bryan et al. [19] evaluated the impact of logging in Northern Borneo (Brunei, Sabah and Sarawak) and estimated that 45,391 km^2^ of intact forest and 68,504 km^2^ of degraded forest remained in 2009, representing 22% and 34% of northern Borneo, respectively. Our numbers for 2010 are of the same order of magnitude: 35,602 km^2^ (17.4%) of intact forest and 74,364 km^2^ (36.4%) of logged forest. Our methods were similar but not identical. As with our analysis, Bryan et al. [19] used a combination of supervised classification and visual interpretation to identify and map logging roads using LANDSAT, and used a buffer distance of only 350 m to estimate the area of forest degradation. Here, based on our observations (see Figure 4), we used a distance of 700 m to define the area impacted by timber harvesting, which likely provides the principle explanation for the differences in estimated intact and logged area between the two studies.

### Relevance for conservation

We show that Borneo has lost forest cover nearly twice as fast as the rest of the world's humid tropical forests [45], in part to create and expand industrial plantations, and in particular oil palm. About 10% of Borneo has become covered in industrial scale monoculture plantations. In addition, Borneo has been intensively logged. The concentration of logging roads found on Borneo is high compared to international standards. Laporte et al. [27], using the same road mapping technique, mapped only 51,916 km (0.03 km of roads per km^2^) of primary logging roads between 1973–2003 in Central Africa (Cameroon, Central African Republic, DRC, Equatorial Guinea, Gabon, Republic of Congo).This road density is 16 times less than our Borneo-wide estimated density. The remarkably high density of logging roads for Sarawak is corroborated by independent observations [19]. Little intact forest remains in that region. But, with 389,566 km^2^ (52.8%) of the island remaining forested, of which 209,649 km^2^ remains intact there is still much hope for biodiversity conservation in Borneo.

In all regions of Borneo it is conversion to plantation development that drives forest loss, and not logging *per se* [17]. A logged forest retains high conservation value [4]. In Indonesian Borneo, the law stipulates that forests in timber concessions are permanent, but the government of Indonesia has bypassed this law. For example, the government re-zoned over 25% of these concessions for plantation development and thus permitted their conversion [42]. In contrast, rates of forest clearance have been lower in Sarawak despite having the highest density of logging roads. Sarawak had developed the most effective techniques to penetrate the rugged forest interior to harvest natural timber, but it did not systematically convert forests to plantations because of the lack of skilled labor [46]. Regional climatic discrepancies between Kalimantan, Sarawak and Sabah may also partially explain the differences we observe. Vulnerability to drought and fire may drive more deforestation in Kalimantan and Sabah than in Sarawak's year-round wetter forests [15], [16], [47].

Protecting logged forests from fires and conversion to agricultural plantations is therefore an urgent priority for reducing rates of deforestation in Borneo. This could be achieved by re-classifying timber concessions in natural forests as protected areas under the IUCN Protected Area Category VI, as many other countries have done [42]. Land use economics, however, drive the rapid conversion of over-logged forests to oil palm plantations where short-term revenues are much higher [48], [49]. Therefore, the law needs to stipulate more strongly that remaining forests need to be kept and conversion of natural forest minimized. There are signs that this is happening, such as the recent re-zoning of a former logging concession into Sebangau National park. Meanwhile oil palm plantations continue to expand, and a much improved understanding is needed on how these potentially conflicting targets can be reconciled and trade-offs minimized [50]. One important factor in this is a more complete valuation of the long-term economic and health values of natural forests managed for timber as well as other services. Especially, the role forests play in sequestering atmospheric carbon and buffer water run-off and thus prevent soil erosion and flooding are important [51], as well as the more traditional potential economic value of timber and other forest products [52].

## Supporting Information

File S1 **Supporting tables and figures.**
(DOC)
