# Supplementary material for: Four Decades of Forest Persistence, Clearance and Logging on Borneo
Source: PLoS One. 2014 Jul 16;9(7):e101654. doi: 10.1371/journal.pone.0101654 (PMC4100734; doi:10.1371/journal.pone.0101654)
Supplement: File S1 — Supporting tables and figures. (DOC) [file pone.0101654.s001.doc]

**Supplementary Figures and Tables**


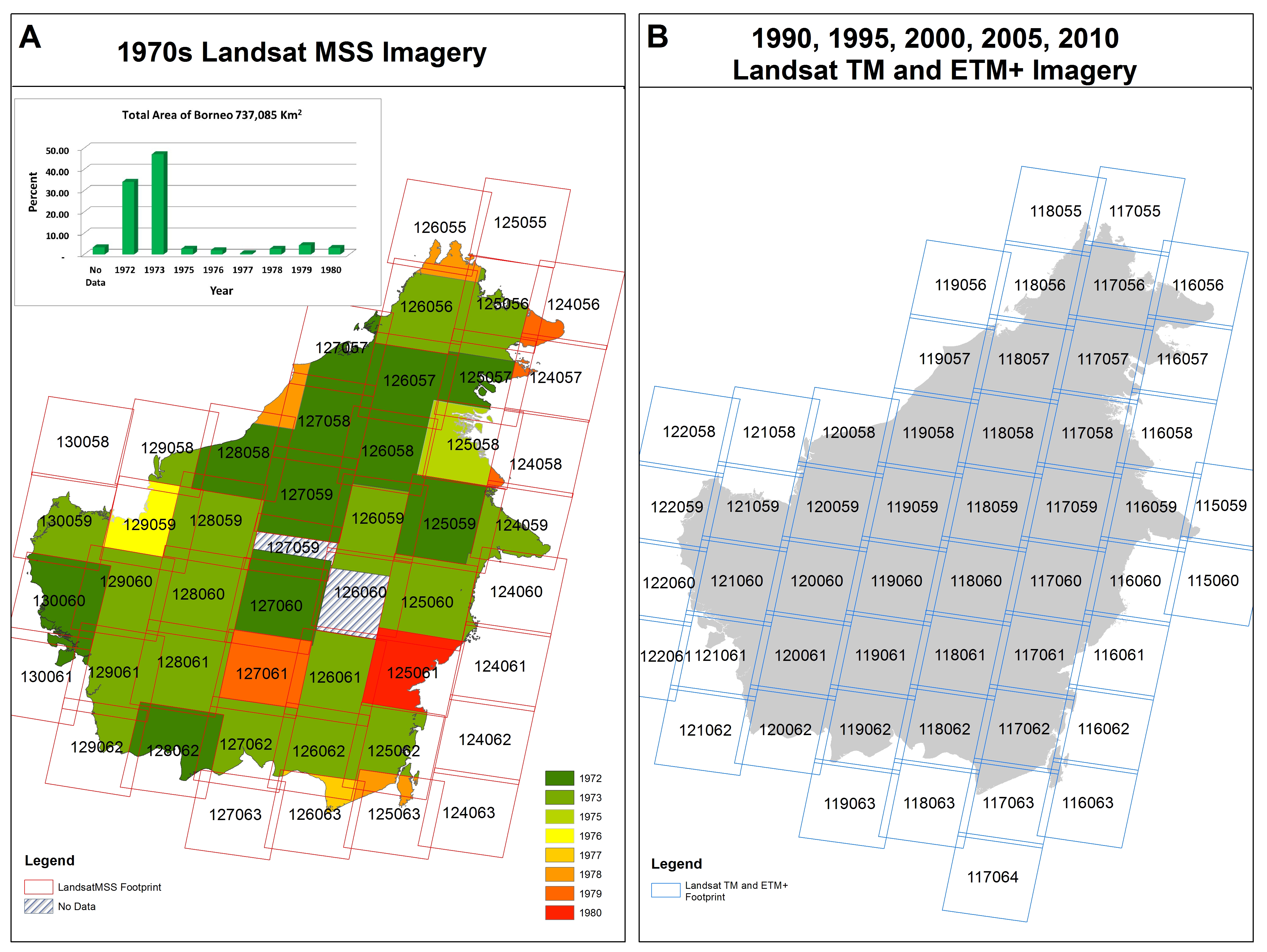


**Figure S1.** A) LANDSAT MSS scenes analyzed to create 1973 forest cover map. ‘No data’ areas had no available imagery, but had full forest cover in 2010 (and therefore also in 1973) because of remoteness. A total a 43 MSS LANDSAT scenes are required to cover Borneo. B) A total of 44 LANDSAT TM and ETM+ scenes are required to cover Borneo. Refer to Table S1 for the detailed list of LANDSAT images used in this study.

**Table S1.** List of all LANDSAT images (MSS, TM, ETM+) used in this study.


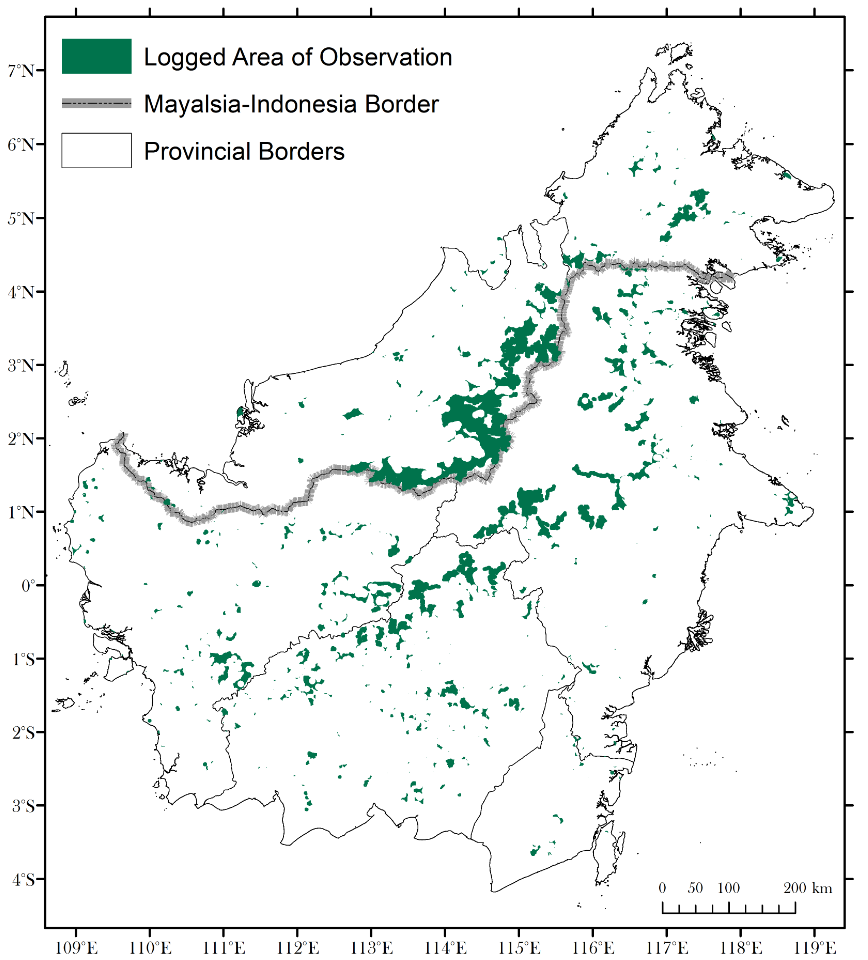


**Figure S2.** Area of observation of the relationship between percent tree cover and distance from select primary logging roads, 2000, by region.

**Table S2.** Area of observation of the relationship between percent tree cover and distance from select primary logging roads, 2000, by region.

|  | Region | | | Total |
| --- | --- | --- | --- | --- |
|  | *Kalimantan* | *Sawawak* | *Sabah* |  |
| Area (km^2^) | 20,981.8 | 15,347.2 | 2,499 | 38,940.6 |
| No. pixels | 390,980 | 285,983 | 46,567 | 725,628 |
| Length of select roads (km) | 6,782.8 | 8,551.5 | 938.4 | 16,336.4 |

**Figure S3.** a) Landsat MSS-based 1973 ‘Forest’ (green)‘Non-forest’ (white) classification; b) corresponding Landsat MSS imagery acquired on 13 July 1973; c) corresponding KH-7 camera imagery acquired on 20 May 1966; d) a view over the KH-7 in maximum resolution.


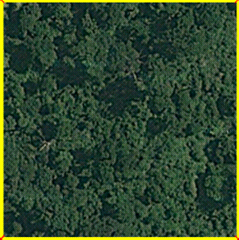


*FOREST (LOGGED)*


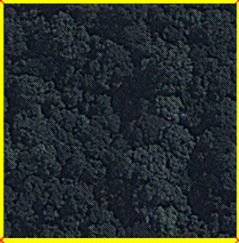


*INTACT*

*MIXED*

*-*

*DIPTEROCARP*

*FOREST*


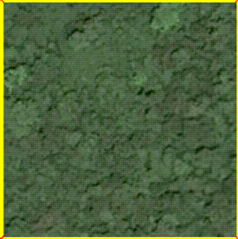


*MANGROVE FOREST*


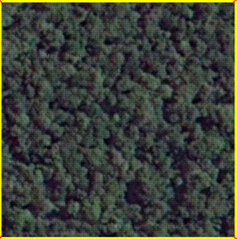


*PEAT SWAMP FOREST*


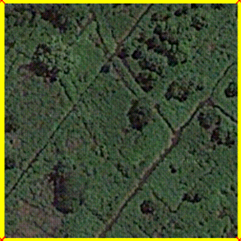

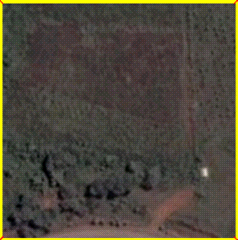


*GARDEN*

*SMALL PLANTATIONS*


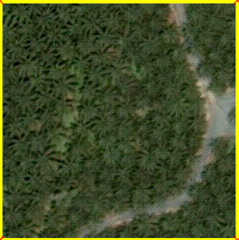


*INDUSTRIAL OIL PALM*

*PLANTATIONS*


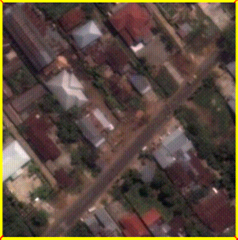


*BUILT UP AREAS*


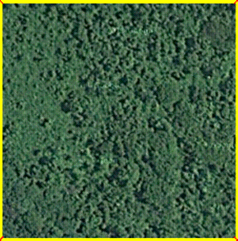


*SCRUB*


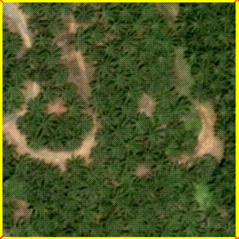

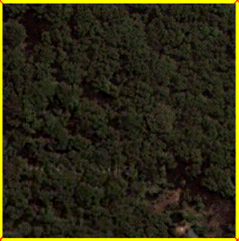


*INDUSTRIAL OIL PALM*

*PLANTATIONS*

*SECONDARY RE-GROWTH*


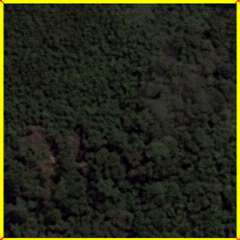


*SCRUB WITH SMALL PATCH*

*OF SECONDARY FOREST*

*SCALE 1:1400*

**Figure S4.** Several QUICKBIRD 60 m x 60 m snapshots (in 1:1,400 scale) for year 2009-2011 showing many subclasses of “Forest”, “Oil Palm”, and “Non-forest”.

**Table S3.** Confusion matrix for the LANDSAT MSS-based 1973 ‘Forest’ ‘Non-forest’ classification validated using high resolution imagery from the KH7 satellite acquired during 1965-68 as the reference. Reference data is in columns. Classification data is in rows. We randomly sampled 322 validation points within the reference area (black areas in Figure 2).

| CLASS | Forest | Non-forest | SUM |
| --- | --- | --- | --- |
| Forest | 194 | 16 | 210 |
| Non-forest | 20 | 93 | 113 |
| SUM | 214 | 109 | 323 |

**Table S4.** Confusion matrix for the for the validation of the ALOS PALSAR and LANDSAT-based 2010 ‘Forest’, ‘Non-forest’, ‘Industrial Timber Plantation, ITP’, and ‘Industrial Oil Palm Plantation, IOPP’ classification using high-resolution imagery (QUICKBIRD and IKONOS) available in Google Earth and acquired during 2009-2011 as the reference. Reference data is in columns. Classification data is in rows. We randomly sampled 1921 validation points within the reference area (black areas in Figure 2)

| CLASS | Forest | ITP | Non-forest | IOPP | SUM |
| --- | --- | --- | --- | --- | --- |
| Forest | 419 | 1 | 38 | 0 | 458 |
| ITP | 0 | 36 | 1 | 0 | 37 |
| Non-forest | 45 | 0 | 1153 | 10 | 1208 |
| IOPP | 0 | 11 | 26 | 181 | 218 |
| SUM | 464 | 48 | 1218 | 191 | 1921 |

**Table S5.** Borneo-wide area estimates (km^2^) of remaining forest in 2010 (intact and logged), forest loss from 1973-2010, industrial oil palm plantations (IOPP) and industrial timber plantations (ITP) realized as of 2010 in the protection, production and conversion zones and by elevation.

|  | Borneo | | | Brunei | | | Kalimantan | | | Sabah | | | Sarawak | | |
| --- | --- | --- | --- | --- | --- | --- | --- | --- | --- | --- | --- | --- | --- | --- | --- |
|  | Prot | Prod | Conv | Prot | Prod | Conv | Prot | Prod | Conv | Prot | Prod | Conv | Prot | Prod | Conv |
|  |  |  |  |  |  |  |  |  |  |  |  |  |  |  |  |
| Total land area |  |  |  |  |  |  |  |  |  |  |  |  |  |  |  |
| 0-500m | 70,588 | 199,085 | 331,614 | 1,104 | 1,801 | 2,680 | 59,864 | 153,155 | 233,986 | 5,072 | 14,685 | 34,614 | 4,548 | 29,444 | 60,334 |
| 501-1000m | 37,358 | 49,324 | 11,724 | 168 | 10 | 8 | 33,527 | 26,829 | 2,349 | 1,994 | 7,357 | 5,029 | 1,669 | 15,128 | 4,338 |
| 1001-1500m | 17,655 | 13,022 | 2,160 | 24 | 1 | 1 | 15,722 | 5,227 | 205 | 1,023 | 1,831 | 1,364 | 886 | 5,963 | 590 |
| 1501-2000m | 2,476 | 1,662 | 366 | 1 | 0 | 0 | 1,979 | 560 | 10 | 261 | 414 | 190 | 235 | 688 | 166 |
| >2000m | 118 | 49 | 1 | 0 | 0 | 0 | 14 | 5 | 0 | 91 | 42 | 1 | 13 | 2 | 0 |
| *All elevations* | *128,195* | *263,142* | *345,865* | *1,297* | *1,812* | *2,689* | *111,106* | *185,776* | *236,550* | *8,4411* | *24,329* | *41,198* | *7,351* | *51,225* | *65,428* |
|  |  |  |  |  |  |  |  |  |  |  |  |  |  |  |  |
| 2010 Intact Forest |  |  |  |  |  |  |  |  |  |  |  |  |  |  |  |
| 0-500m | 33,773 | 50,760 | 29,484 | 831 | 1,292 | 977 | 28,821 | 42,458 | 23,844 | 1,973 | 3,906 | 1,465 | 2,147 | 3,104 | 3,199 |
| 501-1000m | 34,380 | 27,492 | 3,164 | 165 | 8 | 5 | 31,541 | 22,171 | 1,848 | 1,655 | 1,887 | 737 | 1,019 | 3,426 | 574 |
| 1001-1500m | 17,283 | 8,589 | 680 | 24 | 1 | 1 | 15,568 | 5,083 | 193 | 934 | 572 | 322 | 757 | 2,933 | 165 |
| 1501-2000m | 2,408 | 1,267 | 219 | 0 | 0 | 0 | 1,952 | 542 | 10 | 234 | 231 | 104 | 222 | 494 | 105 |
| >2000m | 108 | 42 | 1 | 0 | 0 | 0 | 13 | 4 | 0 | 82 | 36 | 1 | 13 | 2 | 0 |
| *All elevations* | *87,953* | *88,150* | *33,548* | *1,020* | *1,301* | *983* | *77,895* | *70,258* | *25,895* | *4,878* | *6,632* | *2,628* | *4,159* | *9,959* | *4,043* |
|  |  |  |  |  |  |  |  |  |  |  |  |  |  |  |  |
| 2010 Logged Forest |  |  |  |  |  |  |  |  |  |  |  |  |  |  |  |
| 0-500m | 16,216 | 81,573 | 48,303 | 152 | 340 | 316 | 12,668 | 57,842 | 29,099 | 1,598 | 7,235 | 2,800 | 1,798 | 16,156 | 16,088 |
| 501-1000m | 2,133 | 20,222 | 5,176 | 2 | 2 | 3 | 1,335 | 4,083 | 215 | 180 | 4,974 | 1,860 | 616 | 11,163 | 3,097 |
| 1001-1500m | 289 | 4,330 | 1,091 | 0 | 0 | 0 | 122 | 137 | 10 | 42 | 1,221 | 678 | 125 | 2,972 | 403 |
| 1501-2000m | 55 | 388 | 130 | 0 | 0 | 0 | 23 | 17 | 0 | 20 | 179 | 71 | 12 | 192 | 60 |
| >2000m | 5 | 6 | 0 | 0 | 0 | 0 | 1 | 1 | 0 | 4 | 5 | 0 | 0 | 0 | 0 |
| *All elevations* | *18,699* | *106,520* | *54,700* | *154* | *342* | *319* | *14,150* | *62,079* | *29,324* | *1,844* | *13,614* | *5,409* | *2,551* | *30,483* | *19,647* |
|  |  |  |  |  |  |  |  |  |  |  |  |  |  |  |  |
| 2010 planted IOPP |  |  |  |  |  |  |  |  |  |  |  |  |  |  |  |
| 0-500m | 741 | 4109 | 59448 | 18 | 1 | 58 | 266 | 2,799 | 31,514 | 362 | 388 | 15,661 | 96 | 921 | 12,214 |
| 501-1000m | 24 | 88 | 512 | 0 | 0 | 0 | 0 | 0 | 0 | 2 | 12 | 145 | 22 | 0 | 367 |
| 1001-1500m | 0 | 20 | 0 | 0 | 0 | 0 | 0 | 0 | 0 | 0 | 20 | 0 | 0 | 0 | 0 |
| 1501-2000m | 0 | 1 | 0 | 0 | 0 | 0 | 0 | 0 | 0 | 0 | 0 | 0 | 0 | 0 | 0 |
| >2000m | 0 | 0 | 0 | 0 | 0 | 0 | 0 | 0 | 0 | 0 | 0 | 0 | 0 | 0 | 0 |
| *All elevations* | 765 | 4,218 | 59,960 | 18 | 1 | 58 | 266 | 2,799 | 31,514 | 364 | 420 | 15,806 | 118 | 921 | 12,581 |
|  |  |  |  |  |  |  |  |  |  |  |  |  |  |  |  |
| 2010 planted ITP |  |  |  |  |  |  |  |  |  |  |  |  |  |  |  |
| 0-500m | 249 | 1,616 | 8,351 | 0 | 0 | 0 | 94 | 1,548 | 5,805 | 81 | 40 | 1,104 | 73 | 26 | 1,441 |
| 501-1000m | 2 | 18 | 171 | 0 | 0 | 0 | 0 | 1 | 0 | 2 | 18 | 171 | 0 | 0 | 0 |
| 1001-1500m | 0 | 2 | 126 | 0 | 0 | 0 | 0 | 0 | 0 | 0 | 1 | 108 | 0 | 1 | 18 |
| 1501-2000m | 0 | 0 | 2 | 0 | 0 | 0 | 0 | 0 | 0 | 0 | 0 | 2 | 0 | 0 | 0 |
| >2000m | 0 | 0 | 0 | 0 | 0 | 0 | 0 | 0 | 0 | 0 | 0 | 0 | 0 | 0 | 0 |
| *All elevations* | 251 | 1,636 | 8,650 | 0 | 0 | 0 | 94 | 1,549 | 5,805 | 83 | 59 | 1,385 | 73 | 27 | 1,459 |
|  |  |  |  |  |  |  |  |  |  |  |  |  |  |  |  |
| |  |  |  |  |  |  |  |  |  |  |  |  |  |  |  |  | | --- | --- | --- | --- | --- | --- | --- | --- | --- | --- | --- | --- | --- | --- | --- | --- | | 0-500m | 249 | 1,616 | 8,351 | 0 | 0 | 0 | 94 | 1,548 | 5,805 | 81 | 40 | 1,104 | 73 | 26 | 1,441 | | 501-1000m | 2 | 18 | 171 | 0 | 0 | 0 | 0 | 1 | 0 | 2 | 18 | 171 | 0 | 0 | 0 | | 1001-1500m | 0 | 2 | 126 | 0 | 0 | 0 | 0 | 0 | 0 | 0 | 1 | 108 | 0 | 1 | 18 | | 1501-2000m | 0 | 0 | 2 | 0 | 0 | 0 | 0 | 0 | 0 | 0 | 0 | 2 | 0 | 0 | 0 | | >2000m | 0 | 0 | 0 | 0 | 0 | 0 | 0 | 0 | 0 | 0 | 0 | 0 | 0 | 0 | 0 | | *Allelevations* | 251 | 1,636 | 8,650 | 0 | 0 | 0 | 94 | 1,549 | 5,805 | 83 | 59 | 1,385 | 73 | 27 | 1,459 | |  |  |  |  |  |  |  |  |  |  |  |  |  |  |  |

**Prot:** The protection zone includes national parks, nature reserves, wildlife sanctuaries, recreational & hunting parks, and watershed protection reserves. Conversion to agriculture and logging are prohibited.

**Prod:** The production zone comprises areas allocated for commercial logging, but conversion to agriculture is prohibited.

**Conv:** The conversion zone includes regions allocated to industrial plantations, smallholder agriculture, mining, urban areas, and government-sponsored transmigration settlements. Conversion to agriculture is sanctioned.
